# Standardized Classification of Mechanical Ocular Injuries: Efficacy and Shortfalls

**DOI:** 10.14744/bej.2021.01488

**Published:** 2021-09-27

**Authors:** Mahmut Dogramaci, Sevil Karaman Erdur, Fevzi Senturk

**Affiliations:** 1. Department of Ophthalmology, The Princess Alexandra Hospital NHS Trust, Harlow, Essex, UK; 2. Department of Ophthalmology, Istanbul Medipol University Faculty of Medicine, Istanbul, Turkey

**Keywords:** Birmingham classification, classification, mechanical trauma, ocular trauma

## Abstract

**Objectives:**

The aim of this study was to examine the efficacy and the shortfalls of the Birmingham Eye Trauma Terminology classification system for ocular trauma in predicting the visual outcome.

**Methods:**

The records of 256 eyes of 246 patients with a diagnosis of mechanical ocular trauma admitted to the Osman Gazi University Hospital ophthalmology department between 1995 and 2000 were retrospectively reviewed. The zone, type, grade, and pupil status of the injuries were determined according to the Birmingham classification system. Injuries with a good prognosis were defined as injuries that resulted in vision of equal to or better than counting fingers at 1 meter. Fischer’s exact test was used to determine the statistical significance of relationships between the final visual acuity and the initial clinical findings.

**Results:**

Open eye injuries restricted to zone I, those with no afferent pupillary defect, and those graded as 3 or better or classed as type B were significantly associated with a better visual outcome (p<0.05). Open eye injuries that extended to zone III, had an afferent pupillary defect, or were graded as 4 or worse were significantly associated with a poorer visual outcome (p<0.05). Closed eye injuries classified as type B or grade 4 were significantly associated with a poor visual outcome (p<0.05).

**Conclusion:**

The Birmingham classification system for mechanical ocular trauma offers a standardized method for both open and closed eye injuries, however, adding subclasses to type C (injuries with foreign body involvement) could enhance the classification method and help to understand the influence of foreign body properties and sizes on the outcome.

## Introduction

Eye injuries are serious ocular incidents that constitute 10–15% of all ophthalmic diseases with a worldwide incidence of more than 55 million/year (1, 2). In Scotland, the incidence of ocular injuries requiring hospitalization is reported to be 8.1/100,000 persons per year, while in Singapore, it has been reported to be 12.6/100,000 persons per year (2, 3). Similarly, in the United States, the incidence rate was reported to be 13.2/100,000 persons, and in Australia, the rate was reported at 15.2/100,000 persons per year (3, 4). With this in mind, many researchers endeavored to better understand ocular injuries to improve management techniques. However, despite developments into studying the consequences of ocular trauma, it remained difficult up until the introduction of Kuhn’s terminology and Birmingham classification system for ocular trauma (5). The terminology and the classification developed a prognostic model and a scoring system to predict the visual outcome of patients after ocular trauma. The system considered the mechanism of injury, the initial presenting visual acuity, the presence and the absence of afferent pupillary defects, and the zone of the injury. This system was widely adopted by multiple researchers (6-8). Our study aimed to apply ocular trauma classification systems on patients who underwent ocular injuries and were admitted to Osmangazi University to predict their visual outcome and help clinicians and patients in decision-making.

## Methods

The initial clinical findings and the final visual outcomes of 256 eyes of 246 patients who had eye injuries between June 1995 and June 2000 were retrospectively evaluated.

### Inclusion Criteria

• All eye injuries attended Osmangazi University Hospital ophthalmology department between 1995 and 2000

### Exclusion Criteria

The following criteria were excluded from the study:

• Patients with incomplete or missing clinical notes
• Patients who had parts of their treatment continued in other units
• Patients who had follow up periods of <14 days.

The following data were collected from the notes: Date of surgery, type of injury, and laterality, whether the injury was open or closed, mechanism of injury (blunt or sharp), presence of intraocular foreign body, the results of ancillary examinations including computed tomography scans, presence of afferent pupillary defect, the extent of the injury noted during clinical examination, the extent of the injury noted during surgery, the duration of follow-up, and visual acuity at the time of initial presentation and at the final follow-up visit were recorded using decimal values. Cases with a follow-up period of a minimum of 15 days and a maximum of 4 years were included in the study. Kuhn’s terminology and Birmingham classification were applied after the completion of data collection (1, 2).

Eye injuries were first divided into open and closed eye injuries depending on the presence or the absence of a full-thickness defect in the eye wall. Although, posterior to the limbus the eye wall consists of three layers, clinically, the term eye wall was used only to denote the rigid structures of the cornea and the sclera. Eye trauma with full-thickness wounds in the eye wall (corneoscleral) was classified as open eye injuries. Injuries without full-thickness wounds were called closed eye injuries.

Open eye injuries were then divided into four types according to the mechanism of injury. Full-thickness injuries caused by a blunt object were classified as type A (rupture). Single and full-thickness injuries caused by sharp objects in the eye wall were classified as type B (penetrating injury). Injuries with a foreign body in the eye were classified as type C (intraocular foreign body). Double full-thickness injuries in the eye wall that constituted an entry and an exit site were classified as type D (perforating injury).

Both open and closed eye injuries were then divided into five grades according to their visual acuity at the time of initial presentation. Injuries presenting with a visual acuity of 0.5 or better were classified as Grade 1, injuries presenting with visual acuities ranging between 0.4 and 0.2 were classified as Grade 2, injuries presented with visual acuities ranging between 0.1 and counting fingers at 1 m were classified as Grade 3, and injuries with visual acuities ranging between counting fingers at less than 1 m and light perception were classified as Grade 4. Injuries with presented with visual acuities of no light perception were classified as Grade 5.

The injuries were then classified into pupil positive and pupil negative depending on the presence and the absence of relative afferent pupillary defects. The presence of a relative afferent pupillary defect in the injured eye was evaluated as pupil positive, while the absence of relative afferent pupillary defect in the injured eye was considered as pupil negative.

Open eye injuries were also classified into three zones according to the anatomical structures involved. Injuries limited to the cornea (including the corneoscleral limbus) were classified as zone I, injuries involving areas between the corneoscleral limbus and 5 mm posterior to the corneoscleral limbus were classified as zone II, and injuries extending beyond the anterior 5 mm of the sclera were evaluated as zone III. In eyes with multiple open corneoscleral injuries, the zone was defined by the most posterior injury. In perforating injuries, the zone was defined by the most posterior eye wall defect. However, the zone of the injury could be determined at the time of the presentation depending on the initial clinical findings. In our study, the precise zones of the injuries were determined according to the surgical findings as this would have provided more accurate estimation for the zone.

Similar to open eye injuries, closed eye injuries were also divided into types according to the mechanism of the injury. Closed eye injuries caused by blunt objects were classified as type A (contusion). Closed eye injuries caused by sharp edged objects were classified as type B (lamellar laceration), closed eye injuries caused by projectile objects with a foreign body embedded in the conjunctiva and/or the eye wall (corneoscleral) in the absence of full-thickness defect were classified as type C (superficial foreign body), and last, injuries caused by several mechanisms were classified as type D (mixed).

Closed eye injuries were also classified into pupil positive and pupil negative in the same way as open eye injuries. Closed eye injuries were also divided into three zones depending on the anatomical structures involved. Zone I injuries were limited to the external bulbar conjunctiva (sclera and cornea) and classified as zone I. Injuries falling behind the cornea and involving any of the structures within the anterior segment including pars plica but not the pars plana were considered zone II. Injuries involving structures posterior to the posterior lens capsule were classified as zone III injuries.

Finally, both closed and open eye injuries were classified into injuries with good prognosis and injuries with poor prognosis, depending on the visual outcome of the injury at the final follow-up visit. Injuries with a final visual outcome of counting fingers at 1 or better vision were classified as injuries with good prognosis and injuries with a final visual outcome of worse than counting fingers at 1 m were classified as injuries with good visual outcome (3).

Two-sided Fisher’s exact test was computed using SPSS statistical software package version 19 (IBM Corporation, Armonk, NY). This determines the P value of the association of each category of the classification with the final visual outcome category. P<0.05 was regarded as statistically significant.

## Results

A total of 205 male and 51 female patients were admitted to Osmangazi University Hospital with the diagnosis of an eye injury between June 1995 and June 2000. The mean age of males was 30 years (SD 16.5) (range 1–75). The mean age of females was 19.9 years (SD 17.9) (range 1–62). About 47.2% of the patients had injuries to the right eye, and 48.8% had injuries to the left eye. The injuries were bilateral in 4.1% of the patients. Table 1 shows the number of the patients in each age group.

**Table 1.** The number and the percentage of the patients with eye injuries in each age group

Age group	Number of patients	Percentage of patients
0-6 years	28	10.94
7-12 years	23	8.98
13-20 years	46	17.97
21-30 years	56	21.88
31-40 years	44	17.19
41-50 years	30	11.72
51-60 years	14	5.47
60+	15	5.86
Total	256	10.94

The grade of the injury was not determined in eight eyes with open eye injuries and in three with closed eye injuries due to poor cooperation. Table 2 shows the number and the percentage of patients in each group.

**Table 2.** The number and the percentage of eyes within each category determined in accordance with Birmingham classification for eye trauma

Category	Class of the injury (including Type, grade, pupil and zone	Open Eye injuries		Closed Eye injuries	
		Number of patients	Percentage of patients	Number of patients	Percentage of patients
Type of injury	A	40	20	33	58.90
	B	86	43	3	5.40
	C	53	26.50	7	12.50
	D	7	3.50	13	23.20
	E	14	7		
Grade of injury	1	7	3.50	11	19.60
	2	7	3.50	4	7.10
	3	25	12.50	8	14.30
	4	133	66.50	30	53.60
	5	20	10	53	94.60
	Undetermined	8	4	3	5.40
Pupil status	Pupil (-)	117	58.50	44	78.60
	Pupil (+)	54	27.00	11	19.60
	Undetermined	29	14.50	1	1.80
Zone of injury	I	100	50.00	12	21.40
	II	64	32.00	30	53.60
	III	36	18.00	14	25.00
Management details and procedures required	Anti-glaucoma treatment	4	2	13	23.20
	Cataract surgery	53	26.50	9	16.10
	Foreign body removal	32	16	5	8.90
	Pars plana vitrectomy	53	26.50	1	1.80
	Laser photocoagulation	4	2.00		
	Encircling band	17	8.50	2	3.60
	Cryopexy	8	4.00		
	Evisceration	26	13	1	1.80

**Figure 1.**
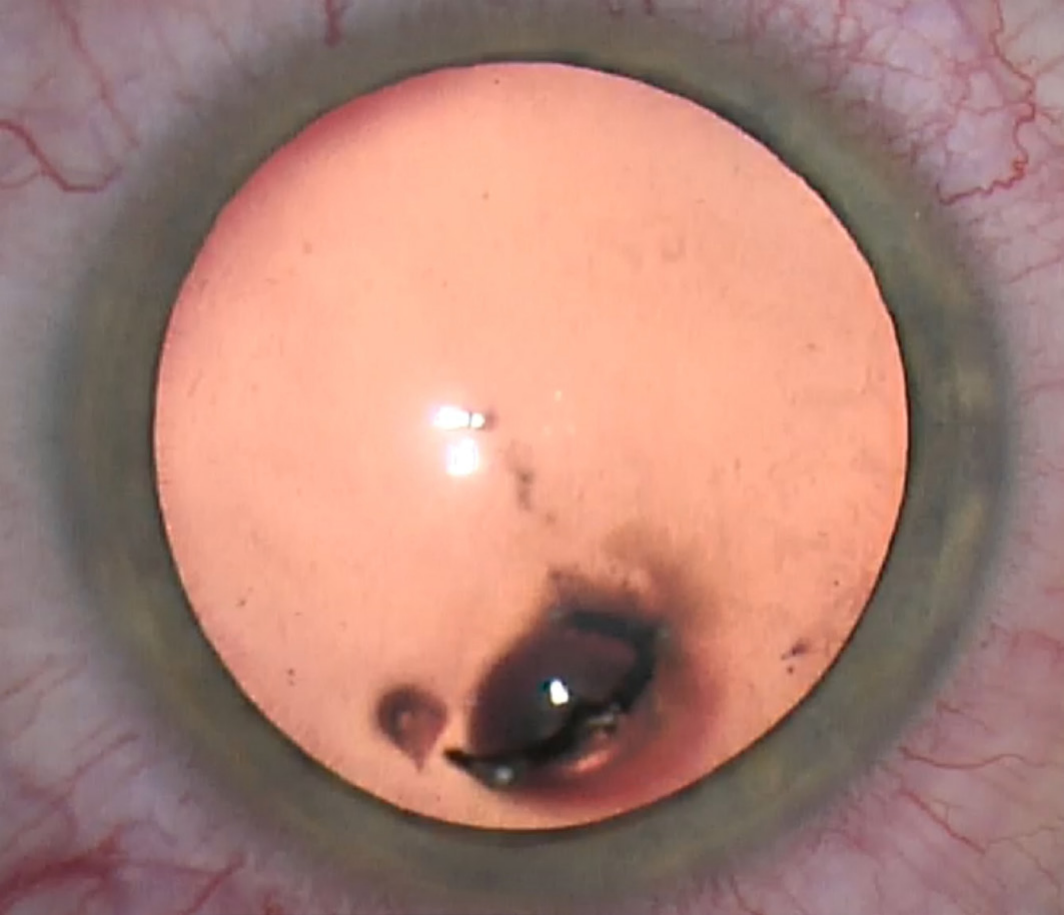
Example of a metal foreign body that caused a full-thickness corneal defect but remained in zone 1.

**Figure 2.**
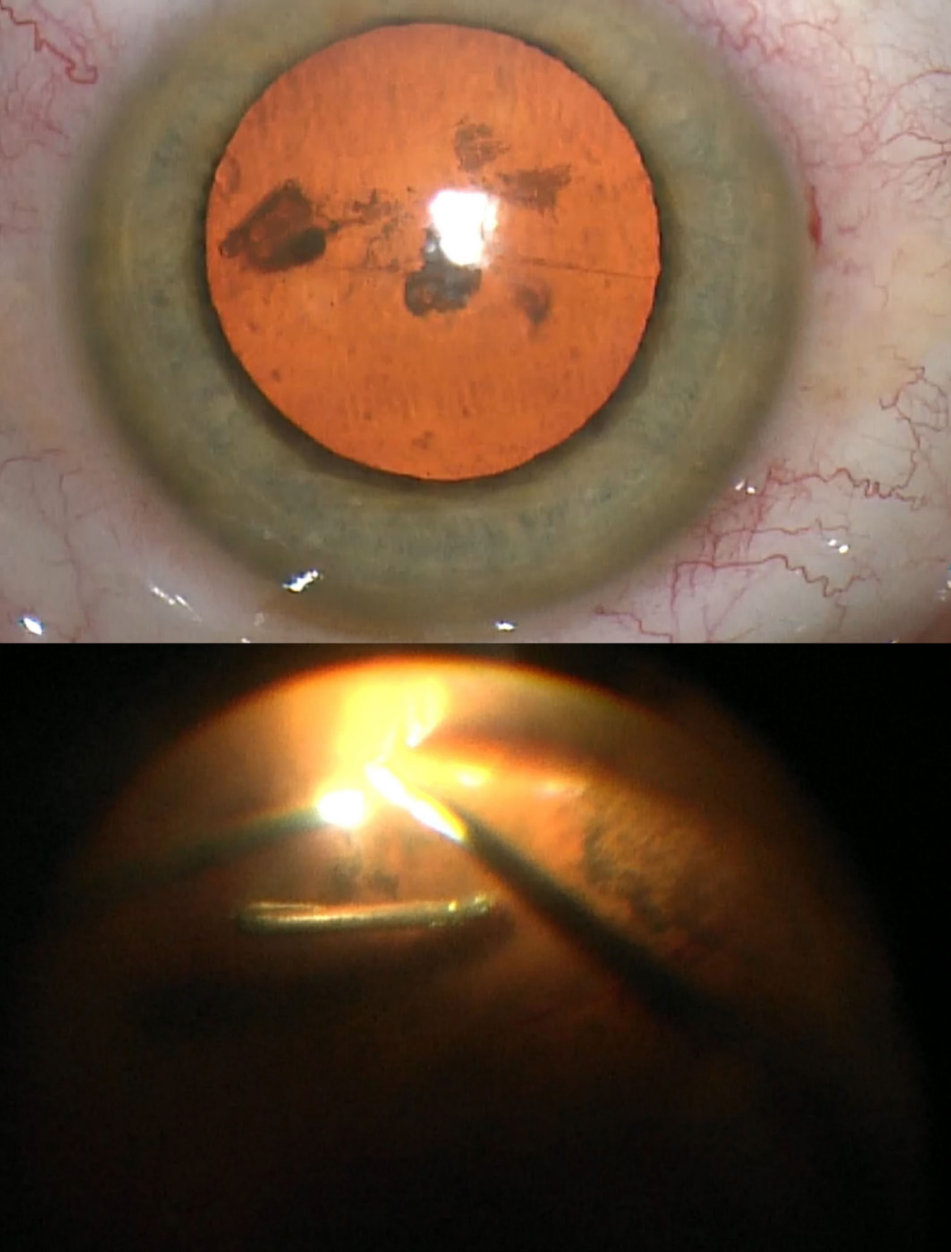
Example of a foreign body that causes full-thickness corneal defect and continued posteriorly and settled on inferior retina

Open eye injuries that were classed as zone I, pupil (-), Grade 3 or better, or type B showed statistically significant association with good visual outcome (p< 0.05). While open eye injuries that were classed as zone III, pupil (+), Grades 4 and 5 showed statistically significant association with poor visual outcome (p<0.05). Closed eye injuries that were classed as type B and Grade 4 showed statistically significant association with poor visual outcome (p<0.05). Table 3 shows the percentage of eyes resulting in good prognosis in each class. Figure 1 shows an example of a metal foreign body that caused a full thickness corneal defect but remained in zone 1. Figure 2 shows an example of a foreign body that causes a full-thickness corneal defect and continued posteriorly settling on inferior retina.

**Table 3.** The percentage of eyes ended up with good prognosis in each class

Category	Open Eye injuries		Closed Eye Injuries	
	Class	% of good prognosis	% of good prognosis
Type of injury	A	38	94
	B	59	100
	C	51	86
	D	29	69
	E	21	NA
Grade of injury	1	100	100
	2	100	100
	3	72	100
	4	43	80
	5	25	NA
Pupil status	Pupil (-)	71	93
	Pupil (+)	9	73
Zone of injury	I	68	100
	II	41	90
	III	11	71

## Discussion

Type E open eye injuries which are caused by multiple mechanisms were associated with the highest rate of poor visual outcome followed by Type D penetrating open eye injuries which were caused with an entry and an exit site. This was in line with other studies that reported bad prognostic outcomes in such types of eye injuries (4-11). Similarly, open eye injuries cause by blunt objects and classified as type A in our study, had higher rate of poor visual outcome compared to open eye injuries caused by sharp objects. Comparable results were reported in the previous studies (4, 7, 12, 13).

Open eye injuries with intraocular foreign bodies which were classified as type C in our study were found to have better prognosis than types A, D, and E. Such reasonable outcomes after well-managed intraocular foreign body (IOFB) have been reported in the previous studies also (6, 7, 12, 14). However, it must be noted that the size and the properties of IOFB could also affect the final visual outcome (15-17). For example, intraocular pellets are known to be associated with worse visual outcomes compared to other types of IOFBs. Poor outcomes in intraocular pellets injuries are mainly due to the contusion associated with such injuries (14, 18, 19). Unfortunately, information regarding the size and the properties of IOFB was not included in Birmingham classification system, therefore, it was not possible to investigate its influence on the outcome.

In line with other studies, open eye injuries that had good visual acuities at the time of presentation did better than those who presented with poor initial visual acuities. Open eye injuries graded as Grades 1, 2, and 3 in our study, had statistically significant better outcome compared to those graded as 4 and 5 (p<0.05). The importance of good visual acuity at the time of presentation in open eye injuries has been reported in the previous studies as well (4, 7, 14, 19-22).

Relative afferent pupillary defect reflects the function of the optic nerve and ganglion cell layer (23). In our study, the final visual acuity in open eye injuries that had no relative afferent pupillary defect at the time of presentation was significantly better than eyes who had relative afferent pupillary defect at the time of presentation (p<0.05). Other studies also showed similar results (4, 13, 15), and one study concluded that the presence or the absence of afferent pupillary defect was the most important factor that determined the final outcome (24). It is unfortunate that pupil examination for afferent pupil defect is often missed or not recorded in patients presenting with eye injuries. Examining the pupil for afferent pupillary defects requires minimal or no cooperation from the patient, and in fact, it could be checked even in an unconscious patient.

The zone of the injury gives an insight into the number of the anatomical structures involved. Our study showed that open eye injuries not extending beyond zone I, had significantly better final visual outcome compared to those that extended to zone II, which, in turn, had significantly better prognosis than those extended to zone III (p<0.05). The previous studies also showed that open eye injuries restricted to the cornea had better final visual outcomes, with only 27–52% of such injuries having a final visual acuity of worse than 0.1, while 30–59.4% of those that extended to zone II had a final visual acuity of worse than 0.5. In comparison, 94% of open eye injuries extending beyond 5 mm of the corneoscleral limbus had a final vision of worse than 0.3 (3, 7, 12, 14, 15, 17, 20, 25-27).

In relation to closed eye injuries, similar to the previous studies, our study also showed that closed eye injuries caused by blunt objects, had a better final visual outcome. This compared to open eye injuries cause by similar objects, with only 6.1–20.8% of closed eye injuries caused by blunt objects had a final vision of worse than 0.7 (28-30). A common example of closed eye injuries caused by blunt objects seen in airbag accidents which is usually limited to zone I. Closed eye injuries that are restricted to zone I could be associated with corneal endothelium damage in 7% of cases, which, in turn, could be the main reason for vision loss (31).

Hyphema, traumatic iritis, iris sphincter rupture, angle recession, iridodialysis, iridodonesis, traumatic cataract, and phacodonesis are common findings in type A closed eye injuries that have extended to zone II. Temporary vision loss is reported in such injuries in 4.3–50% (28-30). It is important to remember that angle recession glaucoma is a common finding in type A closed eye injuries that have extended to zone II. One study found the incidence of angle recession glaucoma in such injuries to be as high as 80.5% (32).

Vitreous base detachment, intravitreal hemorrhage, retinal hemorrhage, commotio retinae, choroidal rupture, and retinal pigment epithelial edema were the main findings in type A closed eye injuries that have extended to zone III. Final visual acuities were reported to be worse than 0.7 in 4.3–53.2% of such injuries (33).

Type B closed eye injuries mainly included lamellar lacerations in the conjunctiva and cornea caused by sharp objects while Type C injuries included superficial foreign bodies. Such injuries are usually treated within outpatients. Therefore, the number of patients that fall into these categories in our study did not reflect the actual total numbers presented to the eye clinic with this kind of injuries. Among those who fell into these categories and at the same time required hospital admissions, were patients who had worse fellow eye injuries, mental health issues. In one case, a patient developed corneal stromal infiltration with hypopyon and the reason for admission was to commence intensive topical treatment.

One of the shortfalls of the study was its retrospective design, however, every effort was made to ensure meticulous collection of data from the notes to enhance the accuracy of the study. Another limitation of the study was its relative short follow-up period of a maximum of 4 years, nevertheless, the authors believe that a 4-year period was long enough to ascertain the final visual acuity in most ocular trauma taking into consideration that some of the consequences, for example ,secondary glaucoma and cataracts could take longer to establish.

## Conclusion

Birmingham classification for mechanical ocular trauma offers a standardized method for both open and closed eye injuries, however, adding subclasses to type C (injuries with foreign body involvement) could enhance the classification method and help to understand the influence of foreign body properties and sizes on the outcome. In open eye injuries, the final outcome is significantly dependent on the zone, pupil status, and the grade of the injury, while in closed eye injuries, the final outcome is significantly related to the type of the injury.

## Acknowledgment

Authors would like to thank Sister Vivienne Padfield RGN, RM, BSC honors, MA for her contribution, read proofing, and correcting grammar and punctuation errors in the paper.

## Disclosures

### Ethics Committee Approval:

Osman Gazi University, applied as requirement for MD Thesis, number 650428, date 27/03/2001.

### Peer-review:

Externally peer-reviewed.

### Conflict of Interest:

None declared.

### Authorship Contributions:

Involved in design and conduct of the study (MD, FS); preparation and review of the study (SKE).
